# Determinants of knowledge about aflatoxin and fumonisin contamination in sorghum and postharvest practices among caregivers of children aged 6–59 months in Kerio Valley, Kenya

**DOI:** 10.1002/fsn3.2502

**Published:** 2021-07-31

**Authors:** Lmeriai Lesuuda, Meshack Amos Obonyo, Maureen Jepkorir Cheserek

**Affiliations:** ^1^ Department of Human Nutrition Faculty of Health Sciences Egerton University Egerton Kenya; ^2^ Department of Biochemistry and Molecular Biology Faculty of Science Egerton University Egerton Kenya

**Keywords:** aflatoxins, children's caregivers, fumonisins, knowledge, postharvest practices, sorghum‐based complementary foods

## Abstract

Stunting among children under five years old is still a problem in many developing countries including Kenya. However, there is little information linking stunting with mycotoxin contamination of complementary foods. The aim of this study was to assess knowledge about aflatoxin and fumonisin contamination in sorghum alongside postharvest handling and storage practices among caregivers of children under five years old in Kerio Valley, Kenya. A cross‐sectional study was conducted to obtain data from 353 randomly selected caregivers of children aged 6–59 months. Qualitative data were obtained through Focus Group Discussions and Key Informant Interviews. Overall, majority of the caregivers of young children had poor knowledge (61.8%) about mycotoxin contamination of food, and poor postharvest handling and storage practices (74.5%). The caregiver's knowledge about mycotoxins was significantly associated with age [(AOR=4.629, (95% Cl: 2.530–8.472), *p* < .001], education level [(AOR=0.275, (95% Cl: 0.088–0.434), *p* = .001], marital status [(AOR=15.187, (95% Cl: 1.830–126.007), *p* = .012], and household monthly income [(AOR=2.623, (95% Cl: 1.550–4.439), *p* < 0,001]. Furthermore, the caregiver's age [(AOR=3.845, (95% Cl: 1.558–9.490), *p* = .003], education level [(AOR=0.196, (95% Cl: 0.088–0.434), *p* < .001], monthly income [(AOR=3.291, (95% Cl: 1.550–6.986), *p* = .002], and knowledge on mycotoxin contamination of sorghum [AOR, 5.428 (95% Cl: 2.855–10.319), *p* < .001] were significantly associated with postharvest handling and storage practices except for marital status [AOR, 3.579 (95% Cl: 0.403–31.775), *p* = .252]. In conclusion, caregivers of young children had poor knowledge about mycotoxin contamination of complementary foods and suboptimal postharvest handling and storage practices of sorghum. This increases the risk of mycotoxin exposure to young children and necessitates mitigation measures including sensitization campaigns and social behavior change communication.

## Graphical abstract

1. The caregivers of young children had poor knowledge about mycotoxin contamination of complementary foods prepared from sorghum.
2. Inadequate knowledge on mycotoxin contamination of food is significantly associated with poor post‐harvest handling and storage practices of sorghum.
3. The main determinants of caregivers’ knowledge and post‐harvest handling and storage practices of sorghum include age, education level and household monthly income.

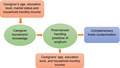

## 1 INTRODUCTION

Mycotoxins are harmful substances produced by some species of fungi that contaminate food material when stored in conditions that favor fungal growth (Obonyo & Salano, 2018). The most significant mycotoxins found in cereals are aflatoxins and fumonisins due to their ubiquitous nature as well as their acute and chronic effects causing death (Probst et al., 2007) or stunting (Knipstein et al., 2015), respectively. There are studies that reported contamination of cereal‐based complementary foods by diverse mycotoxins (Kimanya et al., 2014; Ojuri et al., 2018). This could explain why dietary exposure to mycotoxins is identified as a major cause of undernutrition, morbidity and mortality among children (Paudyal et al., 2017). Children are more sensitive to mycotoxin exposure than the rest of the population due to their underdeveloped immune system, lower body weight, and less acidic stomachs among other factors (Magoha et al., 2014).

In Kenya, sorghum is an important staple food crop for many households especially in the arid and semi‐arid lands (ASALs) (Orr et al., 2016) due to its adaptability to harsh environmental conditions. In this regard, the Kenyan government has continued to develop and promote the production of improved sorghum varieties as a means to improving income and food security (Government of Kenya (GoK), 2018). The Kerio Valley, a semi‐arid area, with unreliable rainfall, chronically experiences food and nutrition insecurity (Elgeyo Marakwet County, 2018) and is currently leading or ranks poorly in the country as far as child nutrition is concerned. A recent study (Kipyego & Mugalavai, 2019) reported a high prevalence of underweight (27%), stunting (67%), and wasting (9%) among children under five years from the small holder farmer households. These rates have remained high despite intervention programs by the county government and other development partners, which included the USAID Feed the Future Accelerated Value Chain Development (AVCD). The AVCD program promoted the production and utilization of sorghum and other drought‐tolerant crops by integrating agricultural and nutrition interventions between 2016 and 2019 (Kiome et al., 2016). It was anticipated that the child nutrition outcomes would improve after the interventions. Therefore, the persistent undernutrition rates necessitated the assessment of underlying factors that contribute to stunting. This is especially important because caregivers of young children from Kerio Valley use sorghum as a sole or major component of complementary foods (in form of porridge) as a common practice (Kiome et al., 2016).

In addition, adequate evidence from neighboring counties (e.g., Nandi County, Kenya) indicates that sorghum is susceptible to aflatoxin and fumonisin contamination at levels above regulatory limits (Sirma et al., 2015). It is thought that co‐occurrence of aflatoxins and fumonisins in complementary foods contributes to stunting in an additive manner (World Health Organization (WHO), 2018) possibly through the toxin‐induced liver and intestinal injury (Knipstein et al., 2015), or impaired immune system function (Leroy et al., 2015). Therefore, this study investigated the factors that determine knowledge about aflatoxin and fumonisin contamination in sorghum and postharvest practices such as handling and storage practices of sorghum among children's caregivers as contributors to possible contamination of complementary foods. There is limited information on the contribution of occurrence and exposure to aflatoxin and fumonisin in sorghum‐based complementary foods to the persistent undernutrition among children under five years in Kerio Valley, which is considered an ultimate goal of this study.

## 2 METHODS

### 2.1 Study area

This study was undertaken in major sorghum growing areas (Emsoo, Endo, and Arror Wards) of Kerio Valley in Elgeyo Marakwet County. The Kerio Valley is semiarid with an annual rainfall of 850‐1000mm and temperature of 40℃‐ 17℃ (Elgeyo Marakwet County, 2018).

### 2.2 Survey design, population, and sampling

A cross‐sectional study was conducted in Kerio Valley, three months (June‐July, 2020) after government lockdown due to the COVID‐19 global pandemic. To obtain the study participants, multistage sampling procedures were adopted. First, Elgeyo Marakwet County was purposively selected being one of the counties where production and utilization of sorghum has been promoted in the AVCD program. Kerio valley was further purposively selected due to the high malnutrition rate reported among children under five years and being the major sorghum‐growing zone in the County, especially in Emsoo ward in Keiyo North, Endo (Marakwet East), and Arror (Marakwet West). The list of the villages growing sorghum in the three wards was obtained from the Ward Agriculture Office, and thirty villages were selected randomly based on probability proportional to population size (PPS) technique. A second sampling frame was generated from the randomly selected villages by enlisting all sorghum growing households with children aged 6–59 months, with the help of the Ward Agriculture Officer, village elders, and lead farmers. Lastly, using Microsoft Excel, simple random sampling was used to select 353 households (Emsoo, = 118; Endo, = 117 and Arror, =118) from the sampling frame generated. The study respondents were the mothers/caregivers of children 6–59 months of age.

### 2.3 Data collection tools

#### 2.3.1 Structured Household Questionnaires

Structured household questionnaires were used to obtain information about respondents’ socio‐demographic information, knowledge about aflatoxin and fumonisin contamination in foods, the health effects of ingesting these toxins in food, postharvest handling, and storage practices of sorghum.

#### 2.3.2 Qualitative data collection

Focus Group Discussion (FGD) and Key Informant Interviews (KII) guides were used to collect qualitative data. One FGD was conducted from each ward among mothers/caregivers of children 6–23 months of age to understand how they handled sorghum and if mycotoxin contamination was of concern when handling children's food. Children aged 6–23 months are nutritionally vulnerable and the introduction of complementary foods that are contaminated with mycotoxins could further impair their growth and development (Alamu et al., 2020). Therefore, it was of importance to have a focus group discussion with the mothers/caregivers of children 6–23 months of age. The FGD participants were selected from villages that were excluded from the household survey. For the key informant interviews, 14 participants including chairpersons for aggregation centers, Ward Agricultural Officers (WAOs), lead farmers, and community health volunteers (CHVs) were selected since they are the frontline agents of change at the community level. They act as intermediaries between researchers and farmers by training and motivating farmers to adapt the good agricultural practices as well as providing free health education training on essential healthcare for mothers and children. The thematic areas of discussion during the in‐depth interviews included food safety issues, aflatoxin and fumonisin awareness, and their management strategies.

### 2.4 Data analyses

All quantitative data were analyzed using Statistical Package for Social Software (SPSS) version 26. Data on caregivers' sociodemographic characteristics, knowledge about mycotoxins, and sorghum postharvest handling and storage practices were analyzed using the chi‐square test. To assess knowledge and practices, every correct answer to the questions was assigned a score of 1 while a score of 0 was given to a wrong answer and where the respondent did not know. Scores on knowledge and practices for each respondent were calculated by summing up the scores attained on each question and the overall score ranked as good or poor. Twelve knowledge questions were asked and thus the scores ranged from 0 to 12. Individuals scoring 7 and below (score below 60%) were categorized as having poor knowledge and scores above 7 as good knowledge (ul Haq et al., 2012; Wang et al., 2015). Regarding the caregivers’ postharvest practice, 18 questions were asked and a total score per individual of less than or equal to 10 (score below 60%) was categorized as poor practices and scores above 10 as good practices (Papagiannis et al., 2020; Wang et al., 2015). Binary logistic regression was used to determine the association between variables of interest. *p* <.05 was considered statistically significant. The qualitative data were analyzed using the thematic content analysis method by identifying similarities, differences, and trends between the individuals and group responses. Common themes that emerged from each individual and throughout the interviews were identified as well as any differences in responses based on the demographics. The findings from the FDGs and KII were then triangulated onto the household survey data to provide in‐depth understanding of the concepts studied.

## 3 RESULTS

### 3.1 Sociodemographic characteristics of the study population

The sociodemographic characteristics of the study participants are shown in Table 1. The household heads for majority of the farmer household were male (93%). More than half (52.1%) of the caregivers were aged 31–45 years and were in monogamous marriage (87.3%). About 45% of farmers had at least primary school education, indicating some level of literacy while more than a third (38%) had a monthly income of less than Ksh. 5,000. All sociodemographic characteristics (sex of the household head (*p* =.034), age of the respondent (*p* =.025), education level (*p* =.001), and monthly income (*p* =.001)) were significantly different in the three administrative wards except for marital status (*p* =.232).

**TABLE 1:** Socio‐demographic characteristics of caregivers’ of children aged 6–59 months in Kerio Valley, Elgeyo Marakwet County

Administrative wards										
Socio‐demographic factors	Total (*n* = 353)		Emsoo (*n* = 118)		Endo (*n* = 117)		Arror (*n* = 118)		χ^2^ value	*p*‐value*
	*n*	%	*n*	%	*n*	%	*n*	%		
**Household head sex**									6.760	0.034*
Male	328	92.9	104	88.1	110	94.9	114	96.6		
Female	25	7.1	14	11.9	7	6.0	4	3.4		
**Marital status**									10.501	0.232
Married monogamous	308	87.3	100	84.7	101	86.3	107	90.7		
Married polygamous	20	5.7	4	3.4	9	7.7	7	5.9		
windowed	9	2.5	5	4.2	2	1.7	2	1.7		
separated	12	3.4	7	5.9	4	3.5	1	0.8		
single	4	1.1	2	1.9	1	0.9	1	0.8		
**Age of the respondents**									14.445	0.025*
18–30	110	31.2	51	43.2	31	26.5	28	23.7		
31–45	184	52.1	51	43.2	62	53.0	71	60.2		
46–60	39	11.0	12	10.2	16	13.7	11	9.3		
Above 60	20	5.7	4	3.4	8	6.8	8	6.8		
**Education level**									30.198	0.001*
None	47	13.3	1	0.8	26	22.2	20	16.9		
Primary	159	45.0	67	56.8	44	37.6	48	40.7		
Secondary	113	32.0	37	31.4	40	34.2	36	30.5		
Tertiary	34	9.6	13	11.0	7	6.0	14	11.9		
**Monthly income**									91.990	0.001*
Less than 5,000	134	38.0	67	56.8	4	3.4	63	53.4		
5,001–10, 000	107	30.3	31	26.3	52	44.4	24	20.3		
Above 10, 000	112	31.7	20	16.9	61	52.1	31	26.3		

* *p* < 0 0.05 significant by chi‐square test

### 3.2 Knowledge about mycotoxin contamination in foods

**FIGURE 1:**
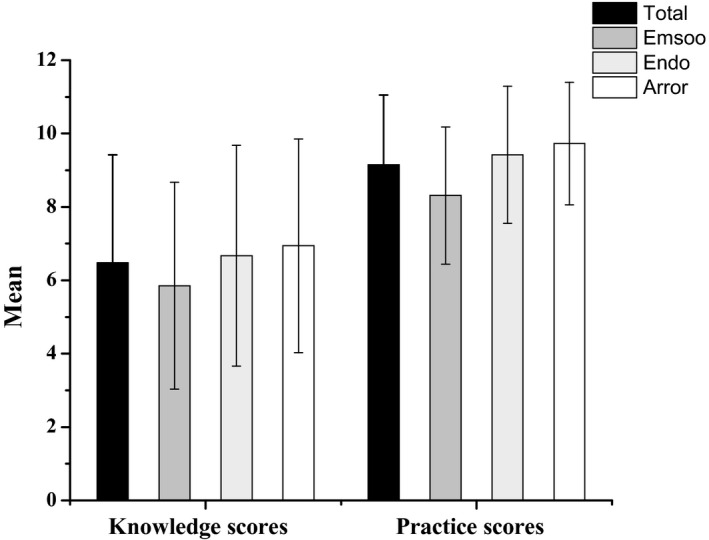
Mean scores of young children caregivers’ knowledge about aflatoxin and fumonisin and postharvest handling and storage practices of sorghum in Kerio valley, Elgeyo Marakwet County. Data are mean ±standard deviations, *p* <.05 significant by analysis of variance (ANOVA)

The average score for knowledge about mycotoxins among the study respondents was 6.48 ± 2.94, out of a possible 12 score, indicating low level of knowledge among caregivers (Figure 1). Of the 353 respondents, 218 (61.8%) were within the poor knowledge score, with more caregivers from Emsoo (71.2%) having poor knowledge scores compared to those from Arror (57.6%), and Endo (56.4%) (Table 2). Although more than half (60.9%) of the respondents had heard about aflatoxins, only 25.2% and 19.8% were familiar with the terms fumonisin and mycotoxins, respectively. Furthermore, out of the caregivers who heard about aflatoxin and fumonisin, 49(13.9%) and 27(33.8%) knew that sorghum grain can be contaminated by aflatoxin and fumonisin respectively. Majority (67.4%) of them knew that consumption of mycotoxin‐contaminated food could lead to adverse health effects such as abdominal pain (55.5%) and diarrhea (22.0%), while 40.8% and 45.9% thought it could also lead to childhood stunting and immune suppression, respectively. The caregivers from Arror (81.4%) were more knowledgeable (χ^2^ =28.939, *p* =.001) of the health effects of mycotoxins consumption in foods was as compared to Endo (63.2%) and Emsoo (57.6%) ward.

**TABLE 2:** Knowledge of caregivers’ of children under five years old about mycotoxins in Kerio Valley, Elgeyo Marakwet County

Administrative wards										
Questions regarding mycotoxins knowledge	Total (*n* = 353)		Emsoo (*n* = 118)		Endo (*n* = 117)		Arror (*n* = 118)		χ^2^ value	*p*‐value
	*n*	%	*n*	%	*n*	%	*n*	%		
1. Heard of mycotoxins? Yes	70	19.8	23	19.5	30	25.6	17	14.4	4.677	0.096
2. Heard of aflatoxin? Yes	215	60.9	66	55.9	80	68.4	69	58.5	4.261	0.119
3. Heard of fumonisin? Yes	89	25.2	24	20.3	36	30.8	29	24.6	3.428	0.180
4. Can mention foods that can be contaminated by aflatoxin? Yes	195	90.7	60	90.9	72	90.0	63	1.3	0.080	0.961
5. Can mention foods that can be contaminated by fumonisin? Yes	57	64.0	17	70.8	20	69.0	20	55.6	1.912	0.384
6. Able to identify spoilt sorghum grains? Yes	327	92.6	108	91.5	111	94.9	108	91.5	1.284	0.526
7. Do you know the causes of mold growth in foods? Yes	321	90.9	109	92.4	103	88.0	109	92.4	1.786	0.409
8. Does consumption of mycotoxins contaminated food has any negative health effects? Yes	238	67.4	68	57.6	74	63.2	96	81.4	28.939	0.001*
9. Do aflatoxins and fumonisins in complementary foods cause:										
1. Stunting? Yes	144	40.8	38	32.2	43	36.8	63	53.4	15.207	0.004*
2. Impaired immunity? Yes	162	45.9	40	33.9	51	43.6	71	60.7	22.974	0.001*
3. Abdominal pain? Yes	230	65.2	68	57.6	74	63.2	88	74.6	11.756	0.019*
4. Diarrhea? Yes	239	67.7	70	59.3	84	71.8	85	72.0	7.657	0.105
Source of information about mycotoxins									16.308	0.178
Mass media (e.g., radio)	86	39.4	24	36.9	34	42.0	28	38.9		
Agricultural extension officers	41	18.8	11	16.9	17	21.0	13	18.1		
Friends/neighbors	41	18.8	10	15.4	17	21.0	14	19.4		
Reading	35	16.1	18	27.7	8	9.9	9	12.5		
Seminars/experts	12	5.5	2	3.1	5	6.2	5	6.9		
Women group	1	0.5	‐	‐	‐	‐	1	1.4		
AVCD‐DT Project	2	0.9	‐	‐	‐	‐	2	2.8		
Knowledge score										
Poor	218	61.8	84	71.2	66	56.4	68	57.6	6.711	0.035*
Good	135	38.2	34	28.8	51	43.6	50	42.4		

* *p* <.05 significant by chi‐squaretest.

### 3.3 Postharvest practices favoring mycotoxin contamination in foods

**FIGURE 2:**
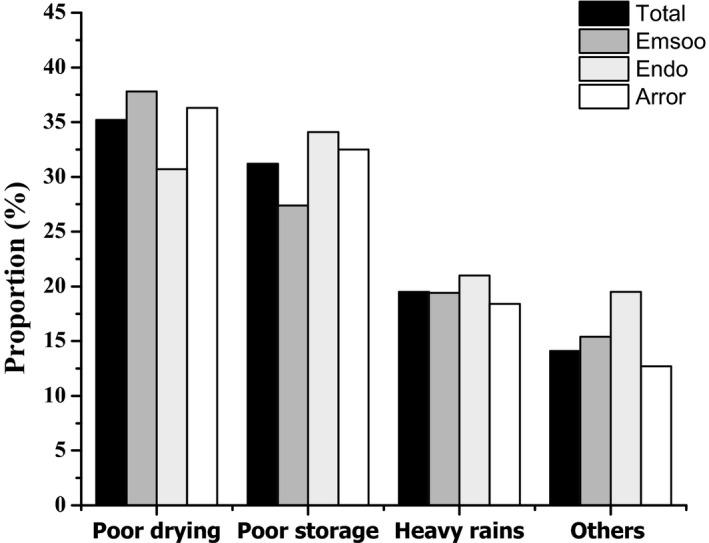
Responses on causes of mold spoilage in sorghum among caregivers’ of young children in Kerio Valley, Elgeyo Marakwet County

Overall, score of the average practices among caregivers was 9.15 ± 1.90, out of 18 total scores (Figure 1). The postharvest practices for majority (75.4%) of the caregivers were poor and diverse (χ^2^ =12.237, *p* =.002) across the three wards. Caregivers from Emsoo ward recorded the highest number of respondents (86.4%) with poor postharvest practices followed by Endo (71.8%) and Arror (70.8%), respectively (Table 3). Overall, less than half of the respondents (35.1% and 31.2%) knew that inadequate drying and poor storage of grains contribute to mold growth resulting in food spoilage (Figure 2). The caregivers from Emsoo (37.8%) and Arror (36.3%) considered poor drying as the main cause of spoilage of sorghum while those from Endo ward (34.1%) pointed out storage as the critical point of contamination. Majority of caregivers dried sorghum after harvest; however, 12.2% left their crops to dry while standing in the field. Among those who dried their grains after harvest (87.8%), duration of drying (χ^2^ =20.807, *p* =.001) and materials used for drying (χ^2^ =15.157, *p* =.004) were significantly different across the wards. Drying sorghum for more than a week was popular in Emsoo while drying grains on bare ground was mainly practiced in Arror ward. To confirm grains dryness, none of the caregivers used a moisture meter but instead, they used traditional methods such as biting (51.0%), looking (24.6%), and touching/squeezing (24.1%).

**TABLE 3:** Practices of the caregivers toward mycotoxins management in Kerio Valley, Elgeyo Marakwet County

Administrative wards										
Households postharvest practices	Total (*n* = 353)		Emsoo (*n* = 118)		Endo (*n* = 117)		Arror (*n* = 118)		χ^2^ value	P‐value
	*n*	%	*n*	%	*n*	%	*n*	%		
1. Dried sorghum after harvest? Yes	310	87.8	100	84.7	102	87.2	108	91.5	2.602	0.272
2. Drying duration										
<7 days	36	11.6	4	4.0	8	7.8	24	22.2	20.807	0.001*
7 days	243	78.4	82	82.0	86	84.3	75	69.4		
>7days	31	10.0	14	14.0	8	7.8	9	8.3		
3. Drying on;										
Bare grounds	199	64.0	67	67.0	57	55.9	75	68.8	15.157	0.004*
Canvas	103	33.1	33	33.0	37	36.3	33	30.3		
Others (Concrete asphalt, rock surface)	9	2.9	0	‐	8	7.8	1	0.9		
4. Methods of checking grains dryness									3.053	0.802
Traditional methods										
Looking at it	87	24.6	27	22.9	27	23.1	33	28.0		
Squeezing/touching	85	24.1	29	24.6	29	24.8	27	22.9		
Biting	180	51.0	62	52.5	60	51.3	58	49.2		
Listening to the sound when winnowing	1	0.3	0	0.0	1	0.9	0	0.0		
Modern method										
Moisture meter	0	‐	‐	‐	‐	‐	‐	‐		
5. Threshing method used										
Hand threshing	340	96.3	116	98.3	114	97.4	110	93.0	4.918	0.086
Use machine/thresher	13	3.7	2	1.7	3	2.6	8	6.8		
6. Removed spoilt panicles before threshing? Yes	353	100	118	100	117	100	118	100	‐	‐
7. Suspect fungal contamination in grains based on;										
Grains discoloration	265	81.8	85	79.4	80	73.4	100	92.6	‐	‐
Off‐smell	152	46.9	56	52.3	38	34.9	58	53.7		
Bitter taste	3	0.9	3	2.8	1	0.9	0	0		
I do not know	4	1.2	2	1.9	1	0.9	0	0		
8. Use of spoilt grains										
Thrown away	114	32.3	32	27.1	47	40.2	35	29.7	18.357	0.019*
Feed livestock	183	51.8	61	51.7	55	47.0	67	56.8		
Making local brew	48	13.6	19	16.1	14	12.0	15	12.7		
Re‐dry and consume	6	1.7	6	5.1	0	0.0	0	0.0		
Sell	2	0.6	0	0.0	1	0.9	1	0.9		
9. Other practices before storage of sorghum grains? Yes										
Grains sorting	111	41.0	33	33.3	37	40.2	42	50.6	‐	‐
Winnowing after threshing	227	83.8	74	77.1	78	84.8	75	90.4	‐	‐
Cleaning of storage area	59	21.8	16	16.7	20	21.7	23	27.7	‐	‐
Fumigation of storage area	19	7.0	6	6.3	6	6.5	7	8.4	‐	‐
10. Type of store used										
Traditional store	285	52.4	53	44.9	61	52.1	71	60.2	11.267	0.080
Modern store	18	5.1	6	5.1	3	2.6	9	7.6		
Living room with improved structure	37	10.5	17	14.4	12	10.3	8	6.8		
Living room without improved structure	131	32.0	42	35.6	41	35.0	30	25.4		
11. Type of storage bags used										
Gunny bags	94	26.6	47	39.8	28	23.9	19	16.1	40.828	0.001*
Hermetic bags	227	64.3	51	43.2	80	68.4	96	81.41		
Sisal bags	23	6.5	14	11.9	7	6.0	2	0.7		
Placed bundle of grains on the bare floor (*No use of bags*)	9	2.5	6	5.1	2	1.7	1	0.8		
12. Stacking of storage bags										
On wood pallets	207	58.6	59	50.0	64	54.7	84	71.2	16.396	0.012*
On the floor	131	37.1	52	44.1	48	41.0	31	26.3		
On the stones	6	1.7	1	0.8	3	2.6	2	1.7		
13. Duration of grains storage										
0–6 months	235	66.6	71	65.3	84	71.8	74	62.7	3.316	0.314
More than 6 months	118	33.4	41	34.7	32	28.2	44	37.3		
14. Sorghum flour storage material										
Plastic bucket	265	75.1	66	55.9	99	84.6	100	84.7	36.830	0.001*
Gunny bag	63	17.8	39	33.1	10	11.9	14	11.9		
Others (Khaki bag, animal skin bag)	25	7.1	13	11.0	8	6.8	4	3.4		
15. Duration of flour storage										
Less than 7 days	319	90.4	100	84.7	104	88.9	115	97.5	11.393	0.003*
More than 7 days	34	9.6	18	15.3	13	11.6	3	2.5		
16. Ferment sorghum flour (Yes)	140	39.7	52	44.1	41	35.0	47	39.8	2.002	0.368
17. Attended training on IYC (Yes)	36	10.2	12	10.2	7	6.0	17	14.4	4.552	0.103
18. Mycotoxins contamination was part of the training (Yes)	9	25.7	‐	‐	6	85.7	3	18.8	17.752	0.001*
Practices score										
Poor	266	75.4	102	86.4	84	71.8	80	70.8	12.237	0.002*
Good	87	24.6	16	13.6	33	28.2	38	32.2		

* *p* <.05 significant by chi‐square test.

Nearly, all the caregivers (96.3%) used manual threshing methods to shell sorghum, and before shelling, they separated spoilt sorghum panicles from the clean ones. Although more than a third (32.3%) of the caregivers threw away the spoilt grains, majority (51.6%) used them as animal feed, prepared local brews (13.6%) while some from Emsoo ward (5.1%) reported using such grains for human consumption. More than half (52.4%) of the caregivers stored their grains in traditional granaries and before grains storage, less than half sorted (41%) their grains, cleaned (21.8%), or fumigated storage area (7%). Majority of the caregivers from Arror (81.4%) used hermetic bags to store sorghum grains compared with those from Emsoo and Endo (χ^2^ =40.828, *p* =.001). Additionally, a large proportion of the caregivers from Arror (84.7%) and Endo (84.6%) wards used plastic buckets to store sorghum flour for preparing children's porridge with majority of them storing the flour for less than a week (χ^2^ =11.393, *p* =.003). Among the small proportion of caregivers (10.2%) who attended training on Infant and Young Child Feeding (IYCF), only nine of them were trained on mycotoxin contamination of complementary foods. More of the caregivers from Endo ward (χ^2^ =17.752, *p* =.001) were trained about mycotoxins during IYCF trainings while none of the IYCF training content included mycotoxin contamination of foods in Emsoo ward.

### 3.4 Factors associated with caregivers’ knowledge about mycotoxin contamination in sorghum and their postharvest practices

The caregivers’ knowledge scores about mycotoxins contamination in sorghum were significantly associated with age [(AOR=4.629, (95% Cl: 2.530–8.472), *p* <.001], education level [(AOR=0.275, (95% Cl: 0.088–0.434), *p* =.001], marital status [(AOR=15.187, (95% Cl: 1.830–126.007), *p* =.12], and household monthly income [(AOR=2.623, (95% Cl: 1.550–4.439), *p* <.001] (Table 4). The postharvest handling and practices of sorghum (Table 5) were also significantly associated with caregivers’ age [(AOR=3.845, (95% Cl: 1.558–9.490), *p* =.003], education level [(AOR=0.196, (95% Cl: 0.088–0.434), *p* <.001], monthly income [(AOR=3.291, (95% Cl: 1.550–6.986), *p* =.002], and knowledge about mycotoxins [(AOR=5.428, (95% Cl: 2.855–10.319), *p* <.001]. Marital status [(AOR=3.579, (95% Cl: 0.403–31.775, *p* =.252)] was not associated with caregivers’ postharvest handling practices.

**TABLE 4:** Factors associated with caregivers’ knowledge about mycotoxin contamination of sorghum in Kerio Valley, Elgeyo Marakwet County

Demographic variables	Knowledge scores (≤7 and >7)		*P*‐value
	AOR	95% Cl	
Age‐group (years)			
18–30 (reference)	1		
>30	4.629	2.530–8.472	<0.001*
Level of education			
No formal education (reference)	1		0.001*
Formal education	0.275	0.129–0.588	
Marital status			
Not married (reference)	1		0.012*
Married	15.187	1.830–126.007	
Monthly average income (in Ksh)			
Less than 5, 000 (reference)	1		<0.001*
More than 5, 000	2.623	1.550–4.439	

Abbreviation: AOR, adjusted odds ratio.

* *p* <.05 significant using binary logistic regression.

**TABLE 5:** Factors associated with postharvesting handling and storage practices of sorghum among caregivers of children aged 6–59 months in Kerio Valley, Elgeyo Marakwet County

Factors	Practices scores (≤10 and >10)		*P*‐value
	AOR	95% Cl	
Age‐group (years)			
18–30 (reference)	1		0.003*
>30	3.845	1.558–9.490	
Level of education			
No formal education (reference)	1		<0.001*
Formal education	0.196	0.088–0.435	
Marital status			
Not married(Reference )	1		0.252
Married	3.579	0.403–31.775	
Monthly average income (Ksh)			
Less than 5, 000 (Reference)	1		0.002*
More than 5, 000	3.291	1.550–6.986	
Mycotoxins knowledge			
Poor (Reference)	1		<0.001*
Good	5.428	2.855–10.319	

Abbreviation: AOR, adjusted odds ratio.

* *p* <.05 significant using binary logistic regression.

## 4 DISCUSSION

The findings of the current study demonstrated that majority of children's caregivers had poor knowledge about aflatoxin and fumonisin contamination of sorghum‐based foods. Poor knowledge may have contributed to poor postharvest handling and storage practices. During focus group discussions (FGDs), caregivers revealed that mycotoxin contamination was not their concern when preparing sorghum‐based complementary foods. Majority of them thought that aflatoxin and fumonisin solely affect maize, another major staple crop in Kenya. These findings corroborate several other studies that have reported that large rural populations in Kenya are only knowledgeable about aflatoxins contamination of maize‐based foods (Njeru et al., 2019; Obonyo & Salano, 2018; Walker & Davies, 2013). Aflatoxin in maize may have received considerable attention in Kenya because of severe aflatoxin contamination of maize that has caused human deaths repeatedly over the years (Probst et al., 2011). Comparable with the findings reported by Njeru et al., (2019), majority of caregivers in this study were not familiar with fumonisins. This could be explained by the current national efforts that are solely focused on aflatoxins, a situation that is similar in most African countries (Matumba et al., 2014).

Sorting and removing visibly defective panicles and grains is an important critical control point for reducing mycotoxin contamination in farm produce. However, visible grain mold may indicate mycotoxins presence, but still very high levels are possible without any noticeable effect on appearance or smell (Ayo et al., 2018). In the present study, caregivers suspected the presence of fungal toxins in sorghum if the grains were discolored, had an off‐smell or bitter taste, and they used these features during panicles or grains sorting. The spoilt sorghum however still ends up in the food chain for majority of caregivers because they mainly used it as animal feed and/or for human consumption. This practice was notable in Emsoo ward, and one of the caregivers said *"Last year, all of my neighbour's produce got spoilt but she refused to dispose off; they consumed all of it."* Lack of knowledge on the health effects associated with mycotoxins (Mboya & Kolanisi, 2014) and food shortage are among the contributing factors for the use of spoilt grains for human consumption and as animals feed (Anitha et al., 2019; Matumba et al., 2016). The study participants showed a general lack of awareness on the health effects associated with mycotoxins on humans. Notably, like their counterparts from Tanzania (Ngoma et al., 2017), a large number of children's caregivers in the present study associated mycotoxins consumption mainly with acute health effects (abdominal pain and diarrhea). For example, a key informant said, “*These women just know about diarrhoea as the only side effect of consuming contaminated foods. Taking Flagyl® stops it*”—this means that farmers mainly associate mycotoxins with diarrhea and they thought the use of Flagyl*®* (an antibiotic‐nitroimidazole) automatically resolves this effect. On the other hand, majority of them could not link chronic health effects such as stunting with aflatoxins or any other toxins possibly because they are long‐term effects.

Poor harvesting and postharvest handling practices are the major factors that promote mycotoxin contamination (Magembe et al., 2016). In the current study, the caregivers had inadequate knowledge on the contribution of these practices on mycotoxin production in grains. For example, majority of them both in FGDs and household survey pointed out inadequate drying and poor storage as the main factors for the development of molds or fungi although other critical factors like length of storage contributed significantly (Gnonlonfin et al., 2013). While a few of the farmers allowed their produce to dry while standing in the field, majority of them sun‐dried sorghum on the bare ground due to the cost of drying materials. These inappropriate practices increase the risk for mycotoxin contamination of the grains. To confirm grain dryness before storage, all farmers used traditional methods such as biting, touch/squeezing, and a combination of visualization and sound tests. Such traditional practices are inadequate and might lead to the storage of grains while still having high residual moisture content (Hell et al., 2008). In the current study, nearly all caregivers threshed/shelled sorghum using manual methods (beating sorghum panicles in a sack or using a traditional pestle (stone) and mortar (hollow tree trunk), with some of them threshing sorghum on bare ground. Manual threshing exposes grains to fungi through kernel breakage and eventually, mycotoxin production (Taye et al., 2018).

Storing properly dried grains in a clean and dry environment as well as controlling storage pests by use of insecticides and hermetic bags can reduce the build‐up of mycotoxins in the store (Abass et al., 2018). In the present study, more than half of the caregivers from Endo and Arror ward used hermetic bags to store sorghum grains attributable to the intervention by different community development projects. Some caregivers, however, highlighted placing sorghum bundles directly on the floor. Worse still, a good number of them placed the storage bags directly on the floor, which may increase fungal growth in stored food commodities due to moisture absorption (Suleiman & Rosentrater, 2015). Storage structures also play an important role in determining the quality of the stored food. Traditional storage structures, such as traditional granaries and living rooms increase the deterioration of grains by mycotoxins due to rodent attacks, insect damage, high temperatures, and humidity (Maina et al., 2016). In this study, over two‐thirds of caregivers stored sorghum grains in traditional granaries and in dwelling houses. Additionally, the material for keeping flour is of critical importance to maintain the quality of the flour (Hemery et al., 2020). Furthermore, the flour for preparing children's porridge was mainly stored in plastic buckets, which are known to retain heat and moisture and easily promote fungal growth and mycotoxin contamination (Mutegi et al., 2013). Sacco et al., (2020) and Selemani (2018) suggested that paper bags could be a suitable packaging material for flours as they found lower fungal count in flour stored in paper bags compared with flour stored in plastic buckets and low‐density polyethylene bags.

Poor mycotoxins knowledge could lead to poor postharvest practices which in turn contributes to mycotoxin production in susceptible food commodities (Makun., 2013). Poor mycotoxins knowledge observed in this study could be attributed to the low number of mycotoxins awareness campaigns in Kerio valley since very few of the study participants have been trained on issues concerning mycotoxins contamination of complementary foods. In addition, the low number of trainings on infant and young children complementary feeding (IYCF), where more than three‐quarters of caregivers did not attend, possibly contributed to the low mycotoxin knowledge, and eventually poor postharvest handling of ingredients for preparing children's food. In general, caregivers in this study received fewer trainings as compared to their counterparts from Tanzania (Katengesya, 2018). Inadequate awareness on issues related to mycotoxins poses a risk of producing and feeding young children with contaminated complementary foods (Katengesya, 2018). For instance, mixing sorghum flour for preparing child's food with more than two other flours such as maize, millet, groundnuts, or cassava was a common practice in the study area. Apart from this practice interfering with the absorption of important nutrients, it could also increase children's exposure to several mycotoxins (Ojuri et al., 2018). Furthermore, it was revealed during FGDs that traditional tools that were used for grains threshing and grinding were not cleaned between each batch of grains. One participant explained that the reason for not washing some of these tools, for example, the hollow tree trunk (*wero konee*) for threshing, was that it would crack. This indicates that caregivers of young children do not have adequate knowledge on the risks of cross‐contamination of children's food.

A remarkable finding from the study is that the older caregivers (≥30 years) were four times more likely to be aware of aflatoxin and/or fumonisin and had good postharvest practices compared to the younger ones. It is possible that the older caregivers have traditional knowledge or may have learned about these toxins through local mass media during aflatoxin outbreaks in Eastern Kenya. For instance, one of the key informants mentioned having heard about aflatoxin from “the Machakos‐outbreak” a part of the Eastern Kenya hotspot (Obonyo & Salano, 2018). The experience caregivers gained over time could also improve their knowledge on mycotoxins and their management practices (Udomkun et al., 2018). Interestingly, the caregivers who attended a formal system of education were less likely to know about mycotoxins or to have good postharvest handling practices. This may imply that knowledge on mycotoxins and management practices are more of transferrable skills (Gichohi‐Wainaina et al., 2020) than what is learnt in school. In the study, very few caregivers obtained information about mycotoxins through reading despite some level of literacy reported which probably indicates the scarcity of written resources, low reading motivation on the side of farmers, or else the materials are too technical for them to understand (Logrieco et al., 2018). Hence, the findings suggest the need to possibly include topics on mycotoxin and food safety in the available infant and young child feeding guidelines as well as in the primary and secondary school curricula. However, finding on the education level contradict previous studies that reported that the literate population had more knowledge on aflatoxin and other mycotoxins than those who have not attended school (Magembe et al., 2016; Matumba et al., 2016; Udomkun et al., 2018). Therefore, more studies are needed to better inform behavior change communication strategies targeted at mitigating mycotoxin contamination of complementary foods.

Another important determinant of knowledge about mycotoxin contamination of foods was the marital status of the caregiver. Similar to findings reported by Magembe et al., (2016), the current study clearly showed that caregivers who are married and living together with their spouse were more likely to be aware of aflatoxin and/or fumonisin than those who are not (single, separated, or widowed). Living together with a spouse might help in sharing information on mycotoxin issues and their management thus contribute to better mycotoxin knowledge. Income is also one of the most salient factors that influence farmers’ perception and awareness of mycotoxins (Redzwan et al., 2012). In this study, a positive relationship was observed between household monthly income and knowledge about mycotoxins and postharvest handling practices of sorghum. The discussions with the caregivers and interviews with key informants suggested the reluctance by sorghum farmers to invest their limited income to learn about or pay for aflatoxin or fumonisin control. This implies that caregivers with less monthly income are less likely to purchase nonfood items such as drying materials, hermetic bags, or radio that will enhance good postharvest practices and mycotoxins’ awareness. Additionally, people with high incomes are likely to be more careful about food and are more willing to pay for food safety than those with lower incomes (Redzwan et al., 2012). This suggests that poverty and lack of sufficient income might contribute to low levels of awareness and knowledge about mycotoxin, leading to high mycotoxin exposure (Leroy et al., 2015) through poor food handling and preparation.

## 5 CONCLUSION

The study demonstrated that caregivers of young children implement suboptimal postharvest handling and storage practices due to poor knowledge about mycotoxin contamination of sorghum‐based foods and their effects on health. Knowledge and postharvest handling and storage practices were associated with the caregiver's age, educational level, and household monthly income. Therefore, to mitigate the occurrence of mycotoxins in complementary foods and across the food value chain; there is a need to increase awareness about aflatoxins and fumonisins among smallholder farmers and young mothers/caregivers.

## CONFLICT OF INTEREST

The authors declare that they do not have any conflict of interest.

## ETHICAL APPROVAL

This study protocols and procedures were reviewed and approved by the Egerton University Research Ethics Committee (ethical code, EUREC/APP/130/2021). Permission to conduct the study was obtained from Kenya National Commission for Science, Technology, and Innovation, relevant authorities at County, Sub‐County, location, and sub‐location level. All study protocols were conducted in accordance with the Declaration of Helsinki recommendations of 1975, revised 2000. Informed consent was obtained from all participants before the interviews. The Kenya Government guidelines and measures on the prevention of COVID‐19 were followed during interviews.

## Data Availability

Due to privacy/ethical restrictions, the data that support the findings of this study are available on request from corresponding author.
